# Sex-specific metamorphosis of cypris larvae in the androdioecious barnacle *Scalpellum scalpellum* (Crustacea: Cirripedia: Thoracica) and its implications for the adaptive evolution of dwarf males

**DOI:** 10.1371/journal.pone.0191963

**Published:** 2018-02-21

**Authors:** Niklas Dreyer, Jørgen Olesen, Rikke Beckmann Dahl, Benny Kwok Kan Chan, Jens Thorvald Høeg

**Affiliations:** 1 Natural History Museum of Denmark, Section for Biosystematics, Copenhagen, Denmark; 2 Natural History Museum of Denmark, Section for Evogenomics, Copenhagen, Denmark; 3 Research Center for Biodiversity, Academia Sinica, Taipei, Taiwan; 4 Department of Biology, Marine Biological Section, University of Copenhagen, Copenhagen, Denmark; CSIR-National Institute of Oceanography, INDIA

## Abstract

Androdioecy (co-existence of hermaphrodites and dwarf males) is a fascinating yet poorly understood phenomenon. The pedunculated barnacle *Scalpellum scalpellum* is an emerging model species for the system. In *S*. *scalpellum*, dwarf males and hermaphrodites are very different in adult morphology (e.g., in feeding structures and reproductive organs), but they share the same larval development with nauplii followed by cypris larvae. Recently, it was found that *S*. *scalpellum* cypris larvae display both genetic and environmental sex determination, but no detailed morphological study has yet investigated how the settled cypris larvae differ subsequent to settlement. This study investigates the morphological aspects of the onset of sex determination in the cyprids of *S*. *scalpellum* by examining their metamorphosis into either dwarf males or hermaphrodites under laboratory conditions. This study emphasizes morphological differences, such as size and shape of primordial shell plates, development of a flexible peduncle and of thoracopods. It was shown that the cypris larvae start to differ already one day after settlement on either a hydroid (leading to hermaphrodites) or an adult hermaphrodite (leading to dwarf males). Dwarf males gradually developed an ovoid body shape and two pairs of circular scutal and tergal primordia. Such cyprids developed neither a carina nor any peduncle or cirri for feeding. The study concludes that the dwarf males of *S*. *scalpellum* are not just hermaphrodites arrested early in development. This entails that dwarf males constitute their own separate developmental pathways and points to *S*. *scalpellum* dwarf males being more specialized than previously stated. Finally, the study compares differences in dwarf male morphology between *S*. *scalpellum* with two other androdioecious species with less specialized dwarf males and use this to discuss evolutionary implications for the adaptive evolution of dwarf males across the Cirripedia.

## Introduction

In his seminal papers on the Cirripedia, Charles Darwin wrote that ‘the diversity in sexual relations appears to me eminently curious (…)’ but ‘regarding the final cause of separation of the sexes and of the existence of complemental males, I can throw no light’ [1]. Since then, numerous papers have elucidated the evolution of sexual systems, and barnacles have been one of the prime models in these efforts [2, 3, 4, 5, 6, 7]. Thoracican cirripedes (stalked and acorn forms) are of particular interest, since they exhibit an array of sexual systems, comprising both hermaphroditic, dioecious and androdioecious species, the latter being a rare condition, where both males and hermaphrodites co-exist within a species [4, 8]. Whenever present, cirripede males are always small (called dwarf males) compared to their female or hermaphrodite partner to which they are permanently attached. Such dwarf males seem to differ widely in structure within cirripedes and studies suggest that dwarf males have played an important role in the evolutionary radiation of the taxon for a number of reasons. First, dwarf males have evolved independently in several lineages and are likely to be advantageous in being located adjacently to the ovipore. Second, they reach maturity faster than their larger partners and survive at higher rates to this stage [5, 6, 7, 8, 9, 10, 11, 12]. Yet, detailed studies of dwarf male structure and function, and especially their development from the settled cypris larva, exist only for a handful of cirripede species [13, 14].

In all cirripedes, the larval phase terminates with a cypris larva, the sole purpose of which is to locate and settle on a suitable substratum [15]. Cirripede cyprids are surprisingly similar in structure, despite settling on a wide variety of substrata and developing into very different animals after attachment [16, 17, 18, 19, 20]. According to taxon, the settled cyprid metamorphoses into such diverse life forms as suspension feeders, a variety of epibionts on e.g. corals, sponges and even cetaceans or some of the most specialized parasites known in the Metazoa [21]. Thus, cypris metamorphosis is a decisive event in the life cycle of any cirripede species. Among pedunculated thoracican barnacles, some lepadomorphan species are androdioecious. In all of these, the males are attached externally on the capitulum of their hermaphrodite partner, which they resemble morphologically in terms of all visible traits such as general shape, number and morphology of shell plates and the presence of a penis [12, 22, 23, 24]. Therefore, it seems that such males could evolve rather easily by simple heterochrony of characters, such as an early maturation of the male organs. A similar case is also exhibited in a few balanomorphan barnacles [25, 26]. The calanticid cirripedes exhibit a more specialized case as dwarf males can be situated on the mantle rim tissue inside the scutal shell plates. They always remain minute and are therefore true dwarf males, but they still resemble their large hermaphrodite partners to a considerable extent by having several shell plates (including a carinal plate) and also small cirri that may possibly be capable of collecting food [11, 22]. Thus, in the Lepadomorpha, Balanomorpha and the Calanticidae there is little if any morphological distinction between dwarf males and early stages of hermaphrodite development. As noted by Darwin [9], the dwarf males of the pedunculated family Scalpellidae seem, in contrast, to be structurally very different from their female or hermaphrodite partners, being reduced sac-shaped forms without cirri and incapable of feeding [12, 13, 25, 26]. This morphology has recently been documented in the androdioecious barnacle *Scalpellum scalpellum* [27], *Verum brachiumcancri* [28] and others [29].

Kühl [30] was the first to investigate the metamorphosis in acorn cirripedes, and Walley [20] extended this to a thorough study on the anatomy of internal structures during cypris metamorphosis. Subsequent studies by Takenaka [31] and Glenner and Høeg [32, 33] elaborated further on the development of single organs and structures. While Høeg et al. [21] provided detailed knowledge on the cypris metamorphosis, information on cypris metamorphosis still only exists for a handful of species [34, 35, 36]. Moreover, none of these studies concern the post-settlement development of dwarf males in thoracican barnacles and very few studies offer a comparative study on the morphology and evolution of dwarf male morphology in androdioecious species [13]. Here, we (1) provide a morphological component to recent sex determination experiments on *Scalpellum scalpellum* [8] using LM and CLSM, and we compare in detail the metamorphosis between hermaphrodite and dwarf male larvae settled under controlled conditions in the laboratory. We (2) specifically focus on whether the dwarf male morphology represents a stage also passed through during hermaphrodite development, or whether *S*. *scalpellum* dwarf males possess specializations particular to their sex. Hermaphrodites and dwarf males differ with respect to functionally important structures used in reproduction and feeding, so we put emphasis on whether differences in developmental patterns are related to these differences. As for addressing the extent to which *S*. *scalpellum* males can be considered specialized, we (3) compare the characters mentioned above with two other androdiecious cirripedes with ascertained dwarf males, *Octolasmis warwickii* (Poecilasmatidae) and *Smilium peronii* (Calanticidae). We evaluate whether the dwarf males in these species represent the same level of specialization as *S*. *scalpellum* dwarf males or whether they are early arrested hermaphrodites. Finally (4), we compare the morphology of other androdioecious species’ dwarf males and discuss the evolutionary implications of our results, including whether the dwarf male metamorphosis is adaptive.

## Materials and methods

### Sampling and maintenance

**Fig 1:**
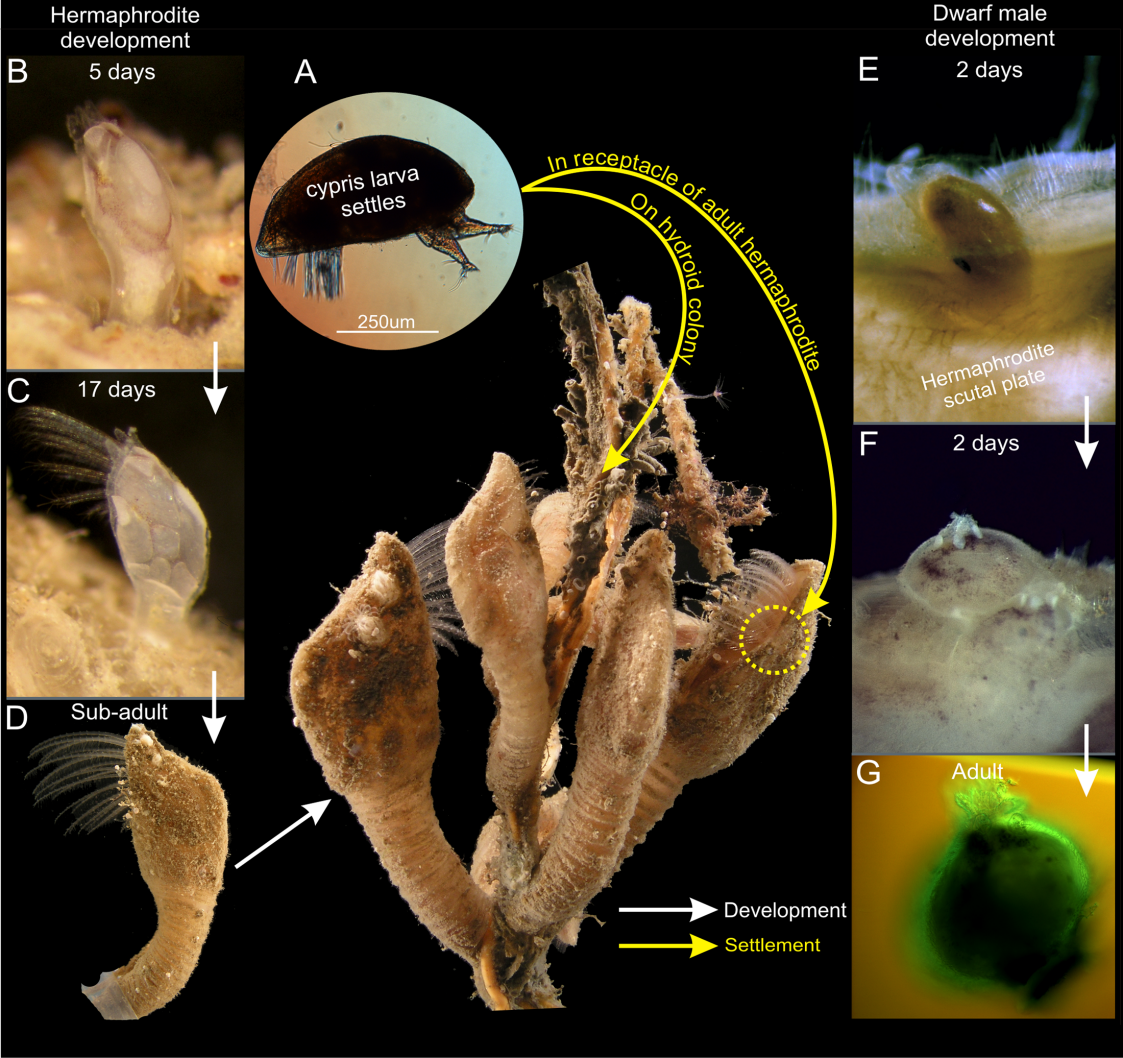
Settlement of larvae in *Scalpellum scalpellum*. Cypris larvae have two options for attachment, and thereafter follow completely different developmental pathways leading to either large, feeding hermaphrodites situated on hydroids (B, C, D) or dwarf males (E, F, G) situated in the receptacles of sexually mature hermaphrodites; yellow arrows indicate settling of cypris larvae; white arrows indicate development.

Specimens of *S*. *scalpellum* were dredged at 30-60m in the vicinity of the Kristineberg Marine Biological Station (KMS) off the west coast of Sweden in 1993. All were attached to hydroid colonies (mostly *Tubularia indivisa*). Maintenance of specimens, collection and rearing of larvae, and settlement experiments with cyprids were undertaken as described in [8, 14, 37]. Specimens developing into dwarf males were obtained from cyprids that were allowed to settle in the receptacles of adult hermaphrodites (see Fig 1). Specimens developing into hermaphrodites were obtained from cyprids settling on *T*. *indivisa*-tubes. Some developing hermaphrodites were also obtained in the experiments designed to yield males, when cyprids instead attached on the external faces of the capitulum or on the peduncles of the adult hermaphrodites. Such cyprids always developed as those attached to the hydroids [8]. Adult hermaphrodites or hydroids were used as settlement substrata by being exposed to free swimming cyprids for well-defined time periods (see below) and thereafter isolated with the larvae that had attached. They were then incubated for increasing time intervals to follow metamorphosis of the cyprids. In this way, the settled and metamorphosing larvae always had a well-defined age. At increasing time intervals after attachment, the metamorphosing specimens were fixed in 2.5% glutaraldehyde in seawater and kept until detailed microscopic examination. Most specimens were allowed to stay *in situ*, where originally attached, until fixation. But as in [8], some specimens were detached and incubated in glass vessels to follow morphological changes more closely *in vitro* and obtain clearly focused videos.

### Material examined

#### *S*. *scalpellum*

We examined a total of 60 hermaphrodites and 58 dwarf males of different ages after initial attachment. For the hermaphrodites, we examined specimens in the following age groups: 1–2, 2–3, 3–4, 4–5, 5–6, 6–7, 7–8, 8–9, 9–10, 10–12 and 14–21 days after initial attachment. Due to scarcity of material, we could only examine males in the age groups 1–2, 2–3, 3–4, and 4–5 days after attachment, but supplemented with numerous specimens of older adult males sampled from the field. These older males were morphologically identical to the day 5 specimens in external morphology. All photos of specimens younger than 2 days originate from live specimens.

#### *Octolasmis warwickii* and *Smilium scorpio*

In total, 25 *O*. *warwickii* adult specimens were obtained from the carapace of the crab *Charybdis spp*. in Bisha Harbor, Keelung, NE Taiwan. Specimens were preserved in 10% seawater based formalin for microscopy. We studied both smaller and larger individuals. Those that had settled on the scutal shell plates of another hermaphrodite can according to recent studies be considered true dwarf males [21, 22]. The dwarf males attached on the large hermaphrodites were dissected to check the mantle cavity for incubated propagules. To check for presence of testis and ovary the bodies of both a very small and a large dwarf male of *O*. *warwickii* were dehydrated in graded ethanol (50%, 70% and 100%) and transferred to 100% Xylene. The samples were then embedded in paraplast, serially sectioned at 8μm and stained in Ehrlich’s haematoxylin and eosin.

Six adult specimens of of *Smilium scorpio* (Pilsbry), with a capitulum length of 21-27mm, were located in the collections of the Naturkunde Museum, Berlin. Bearing registration number 1875, they were labelled Bangkok Behrendt 1897. All specimens had a penis and two of them carried a dwarf male inside the scutal plate. These two specimens were attached peduncle to peduncle forming a group of two. The remaining four specimens carried no males and consisted of a group of two specimens and two solitary ones.

### Microscopy

Most metamorphosing specimens were studied and photographed either in a Leica dissection microscope to obtain overview pictures, in a compound Leica DMRXA microscope fitted with normal and DIC optics or by CLSM as described below. Specimens examined in the compound microscope were fixed in 2.5% glutaraldehyde in seawater. The specimens were then washed in distilled water and transferred to a very weak glycerin solution and incubated for 1–2 days, allowing the residual water to evaporate. This assured a very gradual and complete penetration of the glycerin into the specimens.

CLSM observations were done with auto fluorescence in a Leica DMRXE 6 TL fluorescence microscope equipped with a TCA SP2 AOBS confocal unit and using a 543nm helium/neon laser and a 570μm long pass emission filter. Image stacks were obtained, but the pictures illustrated here are all from single frames. Scanning Electron Microscopy (SEM) was also used but yielded little information because the metamorphosing specimens were covered by the cypris carapace during the early and critical period of development. When attempting to remove the carapace, the specimens would mostly collapse due to tissue porosity.

### Video

Metamorphosing larvae were video recorded in vivo under a dissection microscope either *in situ* or in glass vessels after removal from their hermaphrodite or hydroid substratum (see above). Recording was done in 1993 with a JVC super VHS machine and the tapes later transferred to digital format, analyzed and the final sequences produced using Power Director 13.

## Results

### *S*. *scalpellum*

**Fig 2:**
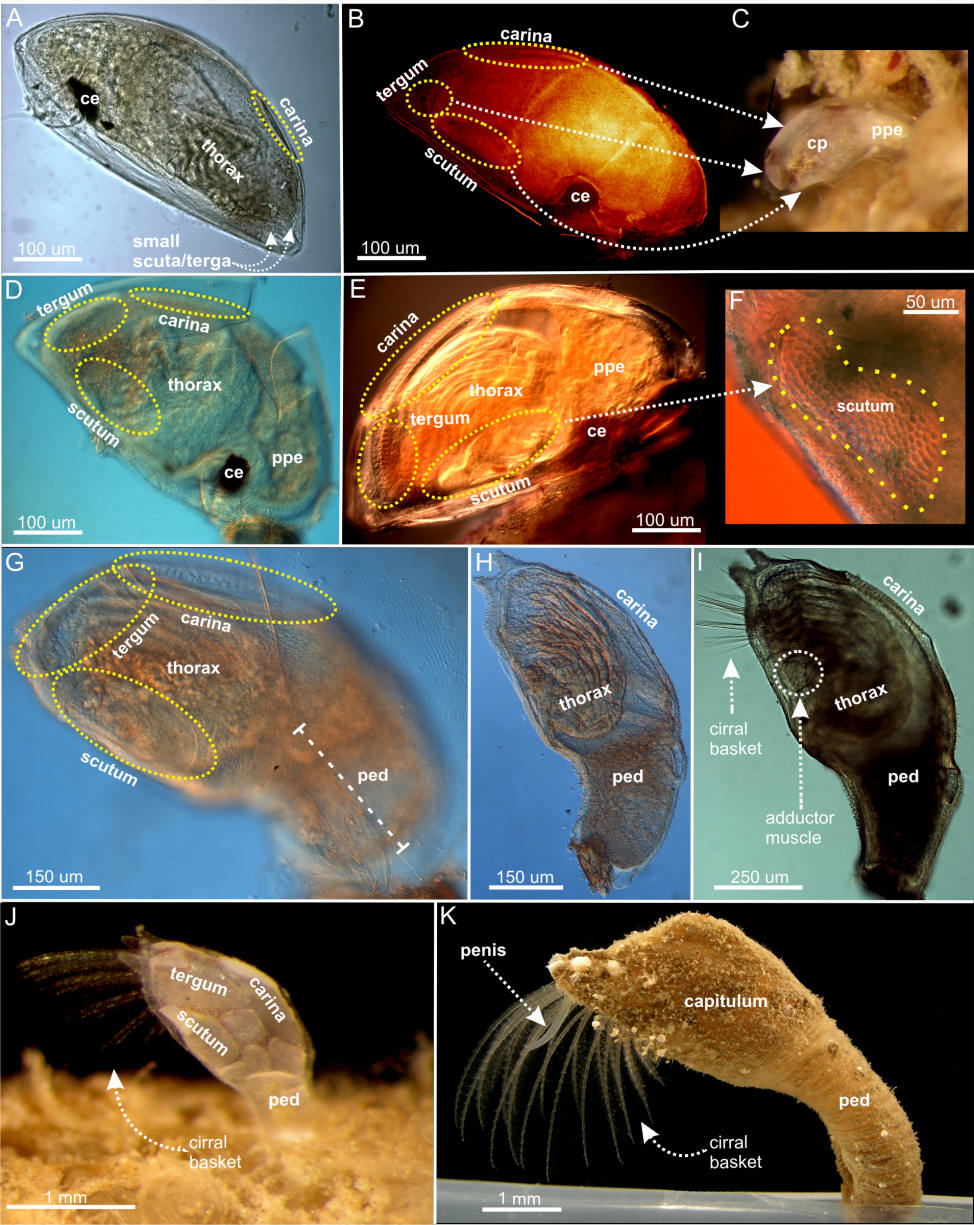
Hermaphrodite metamorphosis in *Scalpellum scalpellum*. Metamorphosing cyprids originally settled on hydroids but removed and incubated in vitro. (A) 1 day after settlement; with only a dorsal carinal primordium, but no other plates. (B) 2–3 days; CLSM, hermaphrodite now with both carina and clearly polygonally shaped primordia of scuta and terga. (C) 2–3 days as in B; specimen photographed still *in situ* on the hydroid. (D) 3–4 days; still enclosed in the cypris carapace, but with incipient peduncle formation at the anterior end (basal). (E) 5–6 days; incipient constriction between peduncle and capitulum; body stance stating to become elevated. (F) Detail of E, showing polygonally shaped scutal primordium (G) 7–9 days; cypris carapace now shed, peduncle has developed so the specimen is raised to near vertical stance. (H) 10–12 days; full armament of shell plates including three latus plates. Plates not calcified and thoracopods not fully functional. Further development of anterior larval tentacles. (I) 14 days; Thoracopods now clearly forming a cirral basket extending from the mantle cavity for feeding. Peduncle slenderer as the hermaphrodite continues to grow. (J) 17 days; juvenile hermaphrodite with calcified shell plates and cirral basket further developed. (K) 21 days; Sub-adult with completion of the capitulum and fully mineralized shell plates. Further development of cirral basket and presence of a transparent penis. Ce; compound eye; cp carapace; ppe pre-peduncle area; pe peduncle; to thorax; ad adductor muscle.

**Fig 3:**
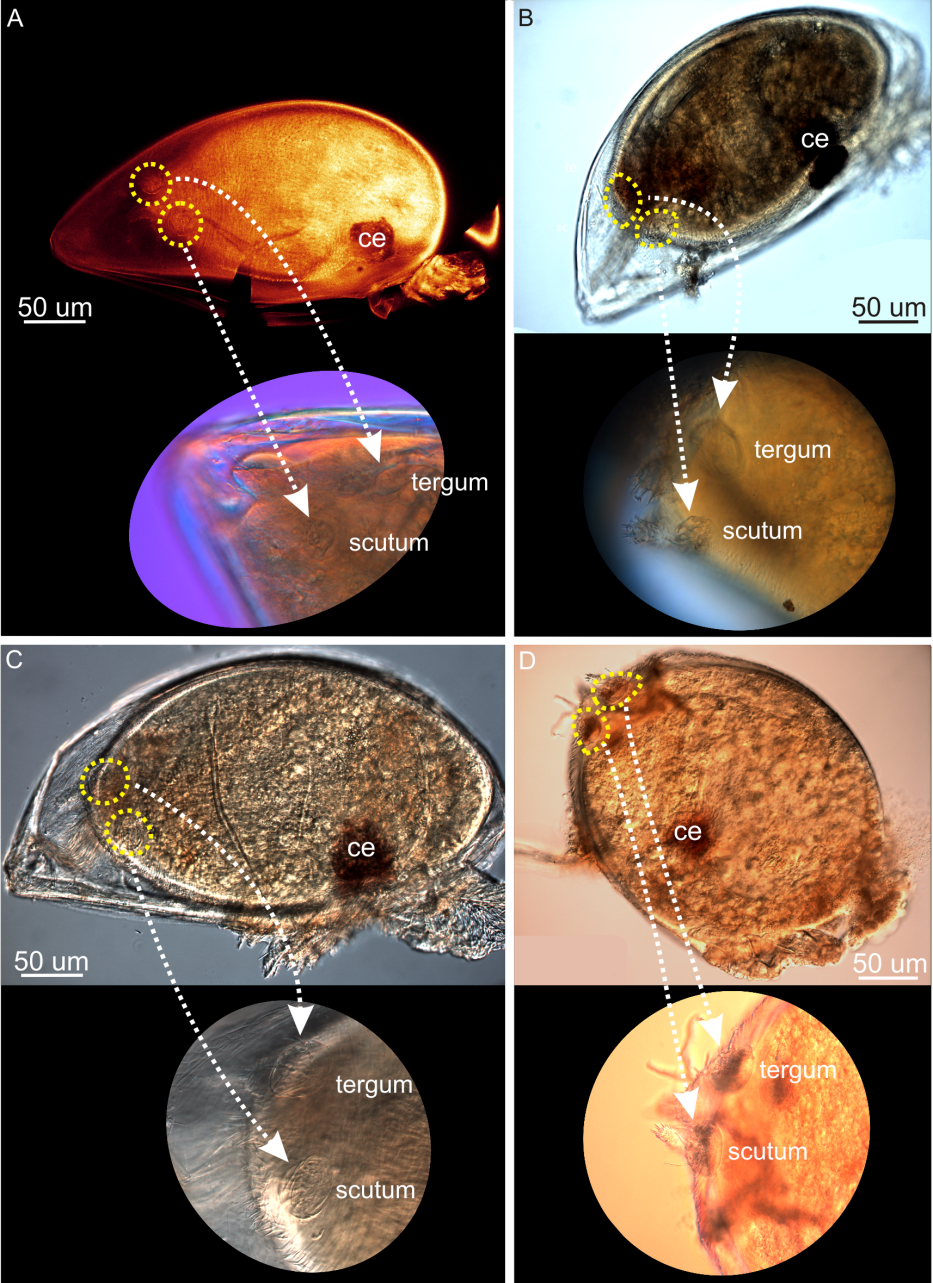
Dwarf male metamorphosis in *Scalpellum scalpellum*. All specimens settled in receptacles but removed and incubated in vitro. (A) 2–3 days after settlement; circular primordial of scuta and terga, but no carina; body shape beginning to assume an ovoid shape. (B) 3–4 days; no carinal plate, scutal and tergal primordia remain small and circular. (C) 4–5 days after settlement. (D) 5–6 days; cypris carapace shed; scutal and tergal primordia still small and circular, no carina; body shape ovoid. ce compound eye; cp carapace.

**Fig 4:**
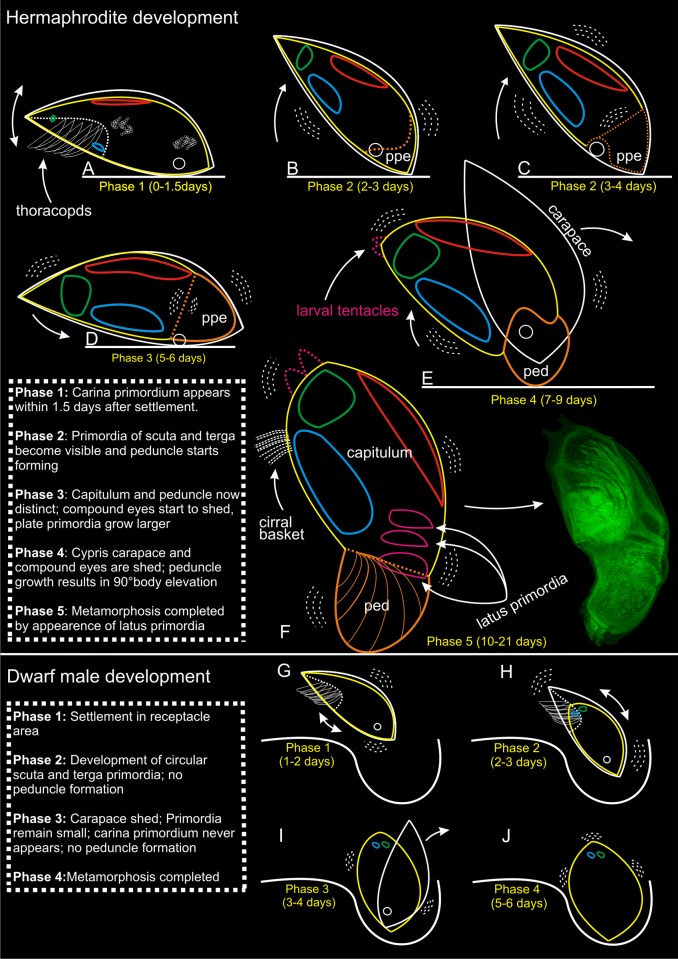
Metamorphosis in *Scalpellum scalpellum*. Summary of key events in hermaphrodite and dwarf male development. Explanatory text on the plate. ppe pre-peduncle area; ped peduncle; blue circles are scuta; green circles are terga; red circles are carina; pink circles are latus.

**Fig 5:**
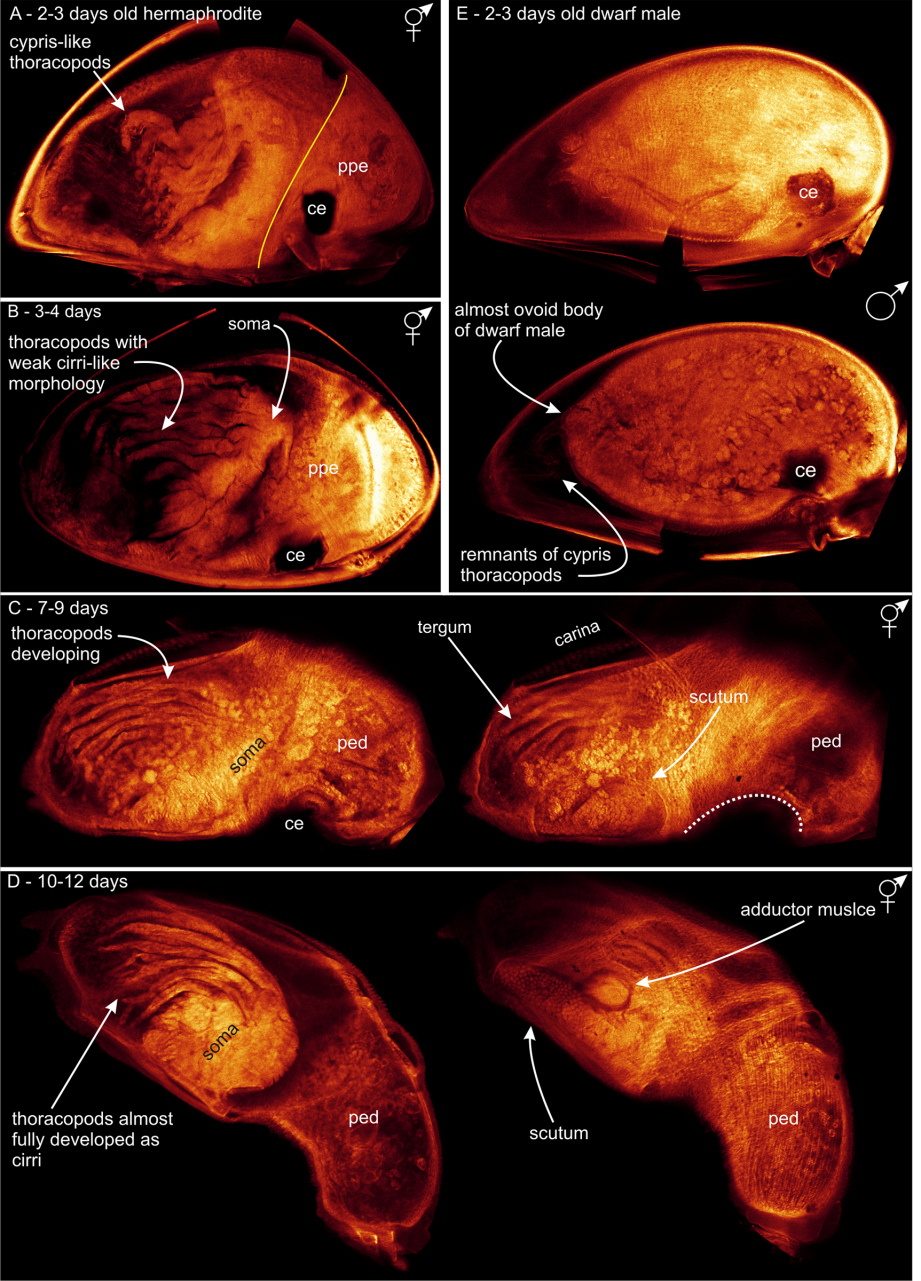
CLSM of metamorphosis in *Scalpellum scalpellum*. (A-D) hermaphrodites; (E) dwarf male. (A) 2–3 days enclosed in cypris carapace but incipient separation into peduncle and capitulum; thoracopods not yet of cirral form. (B) 3–4 days; peduncle, soma and thoracopods better developed; compound eyes moved ventrally and carapace close to being shed. (C) 7–9 days; carapace now shed; very clear separation of peduncle and capitulum; thoracopods further developed. (D) 10–12; hermaphrodite now with the juvenile shape; adductor muscles and soma fully developed but thoracopods not yet cirrus like. (E) 2–3 days old dwarf male; body form almost ovoid, scutal and tergal primordia circular and no carina; remnants of cypris thoracopods visible but no new ones developing. ppe pre-peduncle area; ped peduncle.

S1–S4 Videos provide an overview of the entire sequence of events in the metamorphosis into hermaphrodites and dwarf males. Figs 1–5 document crucial structural details. Table 1 summarizes the most important characters in dwarf males and hermaphrodites for all three species examined.

**Table 1:** Presence/absence (+/-) of key characters in dwarf males of the studied species, *Scalpellum scalpellum* (Scalpellidae) *and Octolasmis warwickii (Poecilasmatidae) and Smilium scorpio* (Calanticidae).

Species/Character	Carina	Scutum, tergum	Peduncle	Cirri	Penis	Ovaries
***S*. *scalpellum***	-	+ ^(1)^	-	-	+ ^(2)^	-
***O*. *warwickii***	+	+	+	+	+ ^(3)^	-
***S*. *scorpio***	+	+	+	+	+ ^(3)^	-

^(1)^ Small and circular while growing large and polygonal as in hermaphrodites;

^(2)^ Male penis organ very different from that in hermaphrodites;

^(3)^ Male penis resembles that in hermaphrodites.

### Morphology of newly hatched cypris larvae

The free-swimming cyprids are approximately 0.75 mm long and 0.30 mm wide. They have elongated, torpedo-shaped bodies covered by a carapace, which is drawn far ventrally on both sides (Figs 1 and 2A) but lacking a mid-dorsal hinge it is not truly bivalved. The two antennules situated anteriorly carry an array of sensory attachment structures. From the thorax situated in the posterior part of the body extends six pairs of biramous and natatory appendages. The general cypris morphology is therefore as in other cirripedes, and in agreement with [8] all larvae had an identical structure with no indication of any sexual dimorphism related to sex. Based on key morphological events, we divide the development of hermaphrodites into five distinct phases and the development of dwarf males into four (Fig 4). The time ranges given for these phases are after cypris settlement by cementation.

#### Hermaphrodite development

**Phase 1 (0–1.5 days)**. The cyprid attaches itself irreversibly by secretion of cement from the tips of the antennules, and during the 0–8 hours period after this event it maintains an orientation with the longitudinal axis parallel to the substratum, as also seen in most other newly settled cirripedes [21, 35]. The developing hermaphrodite body retains a fusiform shape within the cypris carapace (Fig 2A). Posteriorly, covering the dorsal margin of the epidermis, an elongated cuticular primordium of the carinal plate is already clearly seen after one day. More ventrally, cells in the scutal and tergal areas appear dense but they are not yet clearly visible. The compound eyes are located in the antero-lateral region of the carapace.

**Phase 2 (2–4 days)**. This phase marks the real onset of metamorphosis, where several characters unique to hermaphrodites make their first appearance. All five primordial shell plates (paired scuta/terga and carina) are now clearly visible (Fig 2B and 2C). When they first appear, the primordia of the paired scutal and tergal plates are always irregularly polygonal-shaped. The developing hermaphrodite body does not yet fill out the space enclosed by the cypris carapace (Fig 5A and 5B), and, in agreement with [34], has not started to expand in length to any significant degree (Fig 2B). Anteriorly, cells in the future peduncle region begin to compact, and the first signs of a constriction between peduncle and capitulum become visible (Figs 2B and 5A). Posteriorly, two pairs of so-called larval tentacles are now projecting, and the epidermis have begun to separate from the carapace cuticle, indicating the progression of the molting process (Figs 2C and 5A). The compound eyes are still seen in the anterolateral region of the carapace (Figs 2B and 5A). After 3–4 days, the polygonal shell plate primordia of scuta and terga have grown larger and more distinct (Figs 2D and 4C), while the carinal primordium has expanded antero-dorsally. The carapace has not yet been shed but covered inside the hermaphrodite body is now expanding both posteriorly and anteriorly, thus gradually assuming an elongated shape (Figs 2D and 5B). The constriction between the future peduncle and capitulum has become even more pronounced (Figs 2D and 5B). The compound eyes are not yet shed, but have moved more ventrally (Fig 5B), indicating that ecdysis the cyprid cuticle is approaching.

**Phase 3 (5-7days)**. The metamorphosing hermaphrodite starts shedding the compound eyes (Figs 2E and 4D), and there is now a clear separation of the peduncle and capitulum regions. At this stage, the separation of the hermaphrodite epidermis from the cypris carapace has also progressed even further (Fig 2E). At 6–7 days, the stance remains horizontal just as in the newly settled cyprid (Figs 2E and 4D), and the entire body remains enclosed within the cypris carapace, but the formation of a distinct capitulum and peduncle results in a morphology that begins to resemble an adult hermaphrodite. At this stage, the polygonal primordial plates of the carina, terga and scuta have clearly expanded in size (Fig 2G) and the developing thoracopods are now quite distinct (Figs 2E and 5).

**Phase 4 (7–9 days)**. The shedding (ecdysis) of the compound eyes and carapace of the cypris is a gradual process, and its completion marks the transition to phase 4 [29]. Following ecdysis, expansion of the peduncle eliminates the original anterior mantle cavity that in the cypris larva housed both compound eyes and the antennules (Figs 2G, 4E and 5C). At first, this is accompanied by an infolding of the ventral wall behind the antennules, but as the expansion continues, the peduncle rudiment erects the body to an upright 90° position relative to the substratum, thus attaining the stance of the adult barnacle (Figs 2G and 4E). As the primordial plates of the capitulum continue to develop, the carina expands in size along the dorsal midline opposite the ventral aperture between the capitular valves (Fig 5C). At 9 days, the hermaphrodite has further developed the posteriorly projecting larval tentacles, and its primordial plates are now very distinct. Internally, a soma is clearly developing but the thoracopods are not fully functional yet (Fig 5C).

**Phase 5 (10–21 days)**. After 10–12 days after settlement, the hermaphrodite has finally assumed a clearly elongate and slightly curved shape (Figs 2H and 5D). An adductor muscle for opening and closing of the mantle aperture is now present and connecting the scutal plates and the soma is even more distinct, but the thoracopods are still not fully functional (Fig 5D). Forming a small cirral basket, the thoracopods start to emerge from the mantle cavity around 14 days after settlement (Fig 2I) and the specimens start to feed around this stage. As the peduncle slightly stretches as the hermaphrodite continue to grow, individuals exhibit a more elongate and slender peduncle at this stage (2I). The shapes of scuta, terga and carina are now as in an adult and in addition the three pairs of lateral plates have also appeared (Fig 5D). Therefore, the hermaphrodite has now obtained its full armature of plates although none of them have yet become calcified (Figs 2H, 2I, 4F and 5D). During the subsequent development, the cirral basket elaborate further and the shell plates have calcified after 17 days (Fig 2J). 21 days after settlement marks the transition to the sub-adult phase with a further developing cirral basket for feeding and a more calcified and larger shell plate armament now forming a distinct capitulum, which is also seen in adult individuals (Figs 1 and 2K). The presence of a small, transparent penis further indicate that the sub-adult stage has commenced (2K). It remains unclear when the penis is actively used for copulation, but specimens below 7 mm has never been found to carry embryos.

#### Development of dwarf males

**Phase 1 (1–2 days)**. During the first few hours after attachment in the receptacle of a hermaphrodite, no changes take place in the body plan, thus resembling that of a newly settled hermaphrodite. During this period, the cyprid burrows itself deep into the tissue so eventually only the posterior half is exposed to view [13]. Most observations on male development were therefore performed on specimens that had been dissected free and incubated *in vitro*, but comparison showed that events progressed similarly to males that remained *in situ*. With the burrowing completed, the real metamorphosis commences, and at 24 hours the circular primordial of the scutal and tergal plates become distinct at the posterior end of the body, which remains enclosed by the cypris carapace. The shape of the body is still as in a newly settled larva, with the compound eyes clearly visible but no longer moving (Fig 4G).

**Phase 2 (2–3 days)**. The primordia for scutum and tergum is still visible as circular cuticle formations posterior to the weakly developed larval tentacles. The four primordia have not expanded in size as in similarly aged hermaphrodites, but instead retain their initial shape and size into the mature male (Figs 3A, 4H and 5E). There is no trace of a carinal primordial plate, neither at this age nor at a later developmental phase (Fig 5E). The shape of the male body is now beginning to assume its final ovoid shape (Figs 3A, 4H and 5E), but there are no cells clustering at the anterior (basal) end, dovetailing with the fact that no peduncle ever develops in *S*. *scalpellum* males (Fig 5E).

**Phase 3 (3–4 days)**. Contrary to events in the development of hermaphrodites, the carapace is already being shed leaving the two pairs of shell plate primordia completely exposed at the apex, which in deeply burrowed males will often be the only part exposed to view (Figs 3C and 4I).

**Phase 4 (5–6 days)**. The final ovoid shape of the dwarf male has now been obtained (Fig 3D). The posteriorly sited larval tentacles are fully developed around a minute mantle aperture (Fig 4J), but they are rather small compared to the tentacles in the hermaphrodites. Recent SEM pictures of mature dwarf males show that the tentacles have a complex branching structure, and they may possibly be sensory structures [14]. From phase 4 the dwarf males do not change their external structure. Internally, they develop a very large testis and a complexly shaped penis, but it remains unknown exactly when mature sperm becomes available [27].

### O. warwickii

**Fig 6:**
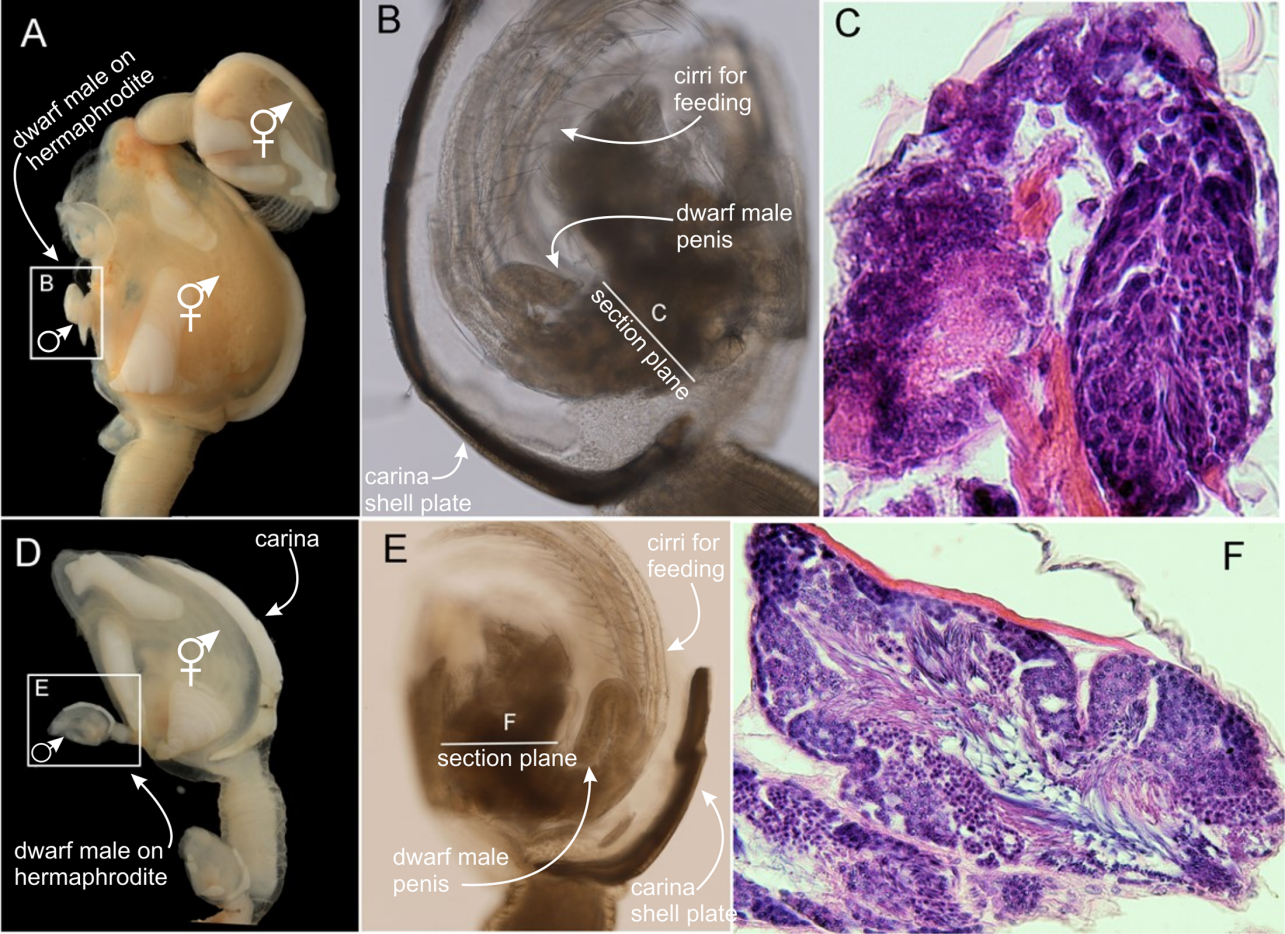
Dwarf males and adult hermaphrodites of *Octolasmis warwickii*. (A, D) Hermaphrodites with dwarf male (squared) attached on the external surface; dwarf male shape identical to their large partner. (B, E) LM of (B) a larger dwarf male and (E) a smaller one, both with peduncle, carinal plate, cirri present and penis. (C, F) Histological section of B and E, showing presence of testes with sperm cells.

Like *S*. *scalpellum*, this is an androdioecious species, whence the adult hermaphrodites contain an ovary, a testis and a penis. Unlike *S*. *scalpellum*, the males do not attach in any preformed pockets in a confined receptacle but invariably settle on the general exterior surface of the hermaphrodites (Fig 1). In accord with recent studies, we only studied dwarf males that had settled on the scuta of their hermaphrodite partner, as these have shown not to be able to develop into functional hermaphrodites [22, 23, 24]. In this lepadomorphan species the hermaphrodites have only five plates, viz., a carina and paired scuta and terga. The males are smaller but externally similar to the hermaphrodites, with a body composed of a peduncle and a capitulum with scuta, terga and carina (Fig 6A, 6B, 6D and 6E). Observations on live specimens also show that the males have fully functioning thoracopodal cirri used for suspension feeding (Fig 6B and 6E). Histological sections of both the small sized and the larger dwarf male showed that no ovary is present, but both had a large testis with mature sperm cells. This indicates that these specimens are in fact functional dwarf males and capable of male function only, and not just juvenile hermaphrodites (Fig 6C and 6F).

### S. scorpio

**Fig 7:**
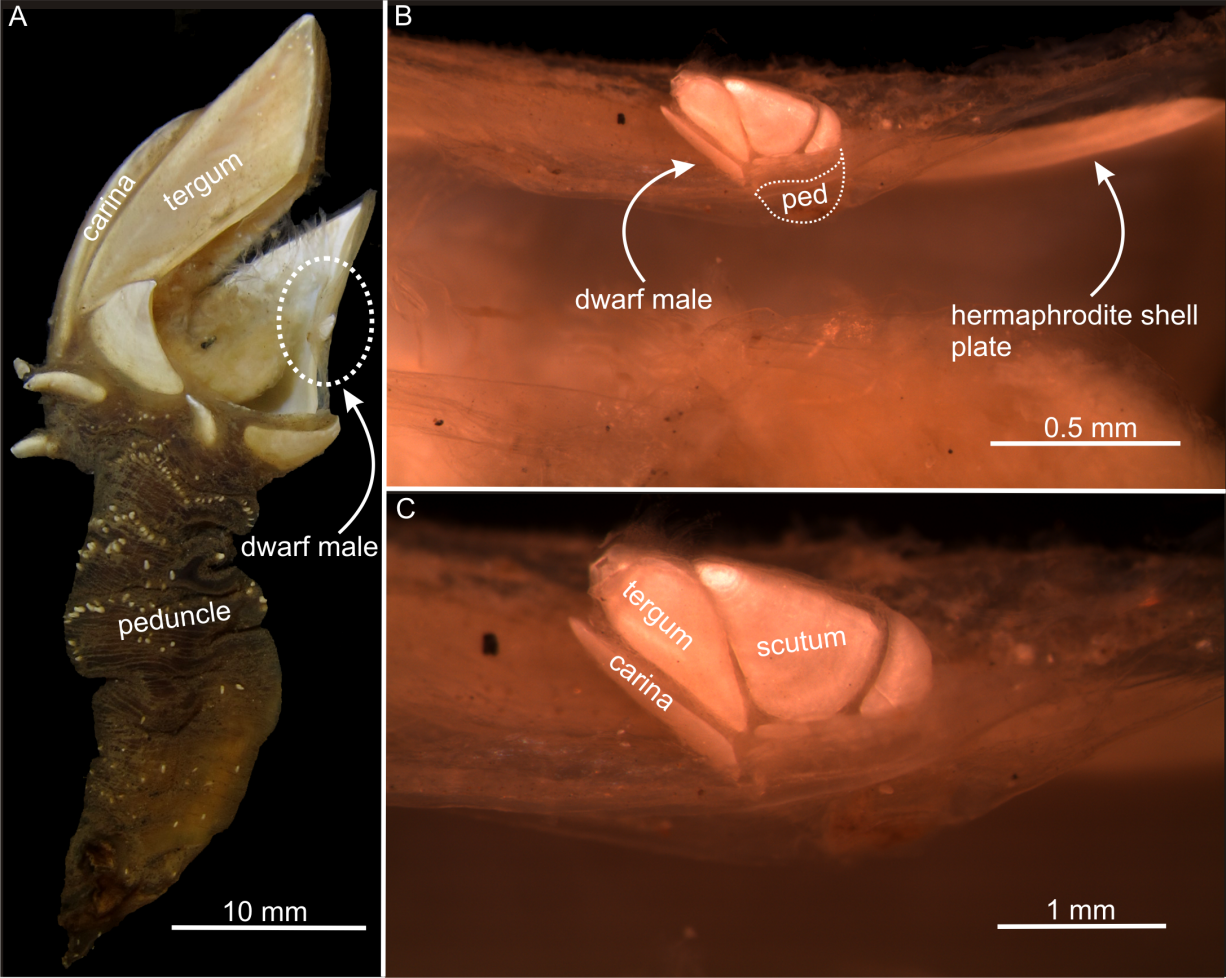
Dwarf males and adult hermaphrodites in *Smilium scorpio*. (A) A hermaphrodite with one scuta dissected exposing left scuta with a dwarf male encircled. (B) Morphology of the dwarf male and its position on the hermaphrodite partner. (C) Close-up of (B), note the presence of almost identical shell plate number and morphology between the dwarf male and hermaphrodite. Ped; peduncle.

This is also an androdioecious species. The dwarf males were attached directly in the cuticle of the mantle rim inside the scutal shell plates, i.e., the same area as seen in *S*. *scalpellum* (Fig 7A, 7B and 7C). The dwarf males are very small, but they possess the same shell plate, penis and cirri morphology as their hermaphrodite partners, with paired scuta and terga and a longer carina in the mid-dorsal line (Fig 7C).

## Discussion

### Are the species examined androdioecious?

The three species examined had dwarf males and hermaphrodites. In all three, the presence of both an ovary and a penis were confirmed in the hermaphrodites [8, 13, 22, 23, 24, 25, 26, 27]. For the dwarf males, serial sections of *S*. *scalpellum* [27] and *O*. *warwickii* (this paper) confirmed the presence of a testis and a penis but no ovary. The *O*. *warwickii* males attached at the margin of the mantle aperture of large hermaphrodites have proven to be true dwarf males. This is established both by their possession of a penis and sperm cells in the testis (Fig 6) and by the direct observation that they never grow into hermaphrodites [24]. Larger individuals are never found in this position in field samples, whereas individuals settled elsewhere (e.g. on the peduncle or carina of the partner as shown in this study) can grow into hermaphrodites. On the other hand, it has been shown that the *O*. *warwickii* males retain the capability to mature into hermaphrodites if they are artificially removed from their settling site on the partner and glued to a neutral substratum [22, 23]. Our material of *S*. *scorpio* originated from a valuable museum collection, so we did not perform histological sectioning to verify the presence of sperm or penis in the males, but males in the Calanticidae are considered to be true dwarf males [13]. In agreement with this, the *S*. *scorpio* dwarf males observed by us are far too small to carry any eggs and, as in *S*. *scalpellum*, they probably have grown little if at all in size compared to the settled cyprid [12, 13]. Furthermore, in the Calanticidae adult hermaphrodites are never attached on the mantle rim of another adult hermaphrodite (pers. observation), adding further proof that the specimens found here are true dwarf males.

### Metamorphosis in *S*. *scalpellum*

In *S*. *scalpellum*, the most distinctive differences during development of hermaphrodites and dwarf males concern the number and shape of the primordial shell plates and the peduncle. Already after two days, the hermaphrodites always have five polygonal, primordial plates, comprising paired scuta/terga and an unpaired carinal plate. In contrast, similarly aged dwarf males have only the paired scuta and terga, which remain minute and oval-shaped, while a carina never appears. Slightly later, the males assume an ovoid shape without any peduncle, while the hermaphrodites assume an elongate shape clearly separated into a peduncle and a capitulum. In males, the oval primordial plates lie around the small sized, semicircular mantle aperture located at the top of the ovoid body and through which they eventually extrude a specialized copulatory tube [27] In hermaphrodites, the plates continue to increase in size, with the carina arming the dorsal side, while the terga and scuta lie along the ventral mantle aperture that is elongated from its first appearance. An additional early difference concerns the appearance of thoracic cirri in the hermaphrodite, whereas no such appendages appear in the males. After shedding the carapace, the non-feeding dwarf males rapidly assume their final adult form and do not increase further in size [14, 36].

In *S*. *scalpellum*, the free larvae are non-feeding, so after settlement the hermaphrodites must soon develop the ability to obtain food by suspension feeding, lest they run out of resources. Both the cirri and the muscular peduncle are functionally a part of the feeding apparatus because the latter is highly flexible, so it can turn the feeding basket in the direction of water currents. None of this is needed in the non-feeding males, and we conclude that the developmental differences point towards a fast development of functionally important structures in either of the pathways, i.e. a feeding apparatus in hermaphrodites and reproductive structures in the male. The thoracopods are clearly visible in newly settled cyprids. In males, these never undergo any morphological development (Fig 5E), while in hermaphrodites they develop gradually along with peduncle (Figs 4D and 5A–5D). In dwarf males, everything points towards a very early attainment of sexual maturity. Males at 6–7 days look exactly as much older specimens in all external features [14], although section series would be needed to ascertain the exact time when mature sperm is present.

### Are dwarf males just early hermaphrodites?

In *S*. *scalpellum*, cypris larvae have two options for settlement and metamorphosis. They can either settle in specialized pockets located in the scutal shell plates of the hermaphrodites, called the receptacles, and develop into dwarf males or elsewhere, mostly on hydroid colonies, and develop into hermaphrodites (Fig 1) [8]. It has recently been shown that *S*. *scalpellum* sports both environmental sex determination (ESD) and genetic sex determination (GSD) [8, 38]. All cyprids are capable of developing into hermaphrodites, but those that that settle in specialized pockets (called the receptacles) located on the upper halves of the scutal shell plates of adult hermaphrodites will invariably develop into dwarf males. This development never occurs elsewhere and suggests that the sex determination mechanism of this species depends on stimuli present only in the receptacle area [14]. It was recently stated that hermaphrodites and dwarf males can be distinguished morphologically after 4–5 days [8], but we can now extend this into males and hermaphrodites starting to diverge morphologically as early as 1–2 days after attachment. Moreover, we find that the final structure of the males exhibits novel features not seen during hermaphrodite development, notably in body shape, in the shape and position of the shell plates and perhaps most significantly in the specialized cuticular tube they use for sperm transfer [27, 28]. We, therefore, conclude that *S*. *scalpellum* dwarf males are not just hermaphrodites arrested in development, but attain their mature state by a unique developmental pathway. In contrast, dwarf males of *O*. *warwickii and S*. *scorpio* (Figs 6 and 7) never exhibit significant features unique to their sex, and we conclude that these males are hermaphrodites arrested in development. The only difference to hermaphrodites is an occasional precocious development of testis and penis and the retarding of female sexual characters. The mode of sex determination and development subsequent to the settlement has not yet been reported for *O*. *warwickii* or *S*. *scorpio*, but since dwarf males and hermaphrodites are near identical in structure they must share virtually the same developmental pathway. The only characters shared between dwarf males of the three species examined in this study are the fusiform shape of the larvae, very early maturation and no display of ovaries with embryos.

### Comparative morphology of dwarf males and attachment sites in other cirripedes

Dwarf males across the thoracican cirripedes (and across all Cirripedia) vary in internal and external morphology [1, 9, 12, 13, 22, 24, 25, 26, 27, 28, 29, 39]. Although dwarf males do vary structurally among scalpellid barnacles, they generally resemble those of *S*. *scalpellum* in having an ovoid or elongated body shape without any peduncle and with at most four minute and apically situated shell plates [9, 12, 13, 40]. They are all very distinctive from their female or hermaphrodite partners, not least in lacking cirri and an alimentary canal, whence these non-feeding scalpellid males depend entirely on the resources allocated to them in the egg. With the possible exception of *Ibla* [13, 29], we therefore argue that scalpellid dwarf males are the most specialized in the Thoracica both regarding their morphology and their location on the partner.

In other thoracican cirripedes the distinction between hermaphrodites and dwarf males is less clear, and based on their morphology and level of specialization, the types of dwarf males can be categorized into four groups [13]. (1) Dwarf males that morphologically resemble their hermaphrodite partners except being smaller and exhibiting a precocious development of male reproductive organs as found in species of *Octolasmis* and *Chelonibia*. Such patterns have also been discovered in *Megalasma striatum* and *Alepas pacifica* [22, 23, 24, 25, 26, 39, 41]. (2) Dwarf males that attach along the mantle aperture but retaining a partial hermaphrodite appearance as seen in many calanticid cirripedes, such as *S*. *scorpio*, *Smilium peronii* and *Calantica vilosa* [13]. (3) Dwarf males that attach in confined areas and morphologically are very unlike their hermaphrodite or female partners. Both males of Scalpellidae and the burrowing barnacles (Acrothoracica) belong to this group [13, 27]. (4) Dwarf males that are nourished by and fully integrated into the female organism in which they function as a “testis”, and where male and female can enjoy a lifelong partnership. Such extreme specialization is found only in the parasitic cirripedes (Rhizocephala) [42].

### Is dwarf male metamorphosis adaptive?

It has recently been shown that dwarf males of thoracican cirripedes have multiple evolutionary origins from hermaphroditic ancestors [5, 12], and this dovetails well with the morphological and biological differences among these males discussed above. We argue that the presence of dwarf males in cirripedes is a secondary evolutionary adaptation, due to special ecological conditions such as small mating group sizes (MGS) and the selective advantage of a much earlier sexual maturation than possible if developing into a large female or a hermaphrodite [5, 7, 9, 12, 24, 26]. Hermaphroditism is most likely plesiomorphic both for scalpellids and perhaps for all thoracican cirripedes, while dioecy evolved from a hermaphroditic ancestor with androdioecy as an intermediate and sometimes even evolutionary stable condition [6, 10]. Scalpellid males are highly specialized in the site where they attach, in their rapid metamorphosis resulting in a very early sexual maturation and in having a minute, yet highly specialized body geared to their sole function of providing sperm while situated in a protected position on their partner animal [7, 14, 29, 39, 40]. For *S*. *scalpellum*, we have here shown that they reach the adult morphology much faster (7–14 days) than the ca. 1 year needed for cyprids settling as hermaphrodites [14, 39]. Undoubtedly, the rapid metamorphosis in males is an adaptation to life in environments, where solitary settlement of a cypris larvae on a hydroid is far less likely to result in producing offspring.

With 250+ described species, the Scalpellidae represent a considerable fraction of the thoracican barnacles, and the family has recently been shown to be monophyletic and originating around the early Cretaceous [9, 11]. Their reproductive systems, including the diverging developmental pathways of males and female-hermaphrodites, the male morphology with its special “penis” and their confinement to special receptacles areas is therefore the result of a very long history that may well have been instrumental in the evolutionary radiation and success of the taxon [8, 14].

### Suggestions for future studies

Cirripedes offer a promising platform for studies on the evolution of sexual systems, and they can serve to illuminate how living species agree with predictions from theory [2, 3, 4, 5, 6, 7, 14, 39]. For this purpose, there now exists a reasonably robust phylogenetic framework, but we are still lacking detailed biological studies of individual species [10, 11, 12], including both population biological data and in-depth studies on sex determination, metamorphosis and sexual maturation. For purely hermaphroditic barnacles, such as *Amphibalanus amphitrite*, there is now a rapidly increasing database of evolutionary-developmental information, while for species with a separate male sex, such information is completely lacking. In *S*. *scalpellum* metamorphosis of virtually identical cyprids begins to diverge hours after attachment and results in completely different organisms. Modern molecular tools, including transcriptomics, could undoubtedly provide crucial insight into how these two developmental pathways are controlled. Furthermore, a comparison with events in both lepadomorphan species with males and purely dioecious scalpellids would be highly interesting, culminating in a more complete understanding of the genetic level basis of cirripede sexual systems evolution.

## Supporting information

S1 Video Hermaphrodite development in *Scalpellum scalpellum*. *In situ* settlement and development on hydroids.
(ZIP)

S2 Video Hermaphrodite development in *Scalpellum scalpellum*. Detached hermaphrodites developing *in vitro*.
(WMV)

S3 Video Dwarf male development in *Scalpellum scalpellum*. (ZIP)

S4 Video Comparison of hermaphrodites and dwarf males developing *in vitro*. (ZIP)
